# Better Multi‐Breed Genomic Predictions for Tropical Bull Fertility Using a Breed‐Adjusted Genomic Relationship Matrix

**DOI:** 10.1111/jbg.70052

**Published:** 2026-04-22

**Authors:** Antonio Reverter, Pâmela A. Alexandre, Marina R. S. Fortes, Laercio R. Porto‐Neto

**Affiliations:** ^1^ CSIRO Agriculture & Food St. Lucia Queensland Australia; ^2^ School of Chemistry and Molecular Bioscience The University of Queensland St Lucia Queensland Australia

**Keywords:** Brahman, fertility, genomic prediction, multi‐breed, percent normal sperm, Santa Gertrudis, scrotal circumference, sheath score, tropical beef

## Abstract

The pressing requirement for agricultural systems to adopt climate change adaptation strategies specifically designed for tropical environments underscores the significance of implementing sustainable bull breeding practices in beef cattle operations. We consider alternative approaches to generation of single‐ and multi‐breed genomic predictions for a population of Brahman (*N* = 1051), Santa Gertrudis (*N* = 929) and UltraBlack (*N* = 844) bulls with genotypes at high‐density and phenotypes for scrotal circumference (SCC; mean ± SD = 31.82 ± 4.32 cm), sheath score (SHS; 3.70 ± 1.98) and percent normal sperm (PNS; 63.34% ± 28.22%). We examined five genomic prediction models: three single breed and two multi‐breed. The later contained a multi‐breed genomic relationship matrix computed without (GRM_u) or adjusting (GRM_a) for breed‐specific allele frequencies. Bias, dispersion and accuracy of the genomic predictions across the five models was computed based on cross‐validation and using the LR method. The elements of the multi‐breed GRM_u revealed anomalies including a multi‐modal distribution of diagonal and off‐diagonal elements with all diagonal values above one (range: 1.022 to 1.524) and averaging 1.163. Instead, GRM_a values were consistent with expectations: diagonals with a single mass around one (range: 0.844 to 1.391) and off‐diagonal values with a single mass around zero. Estimates of heritability (*h* ^2^ ± SE) with the GRM_u model were 0.501 ± 0.037, 0.458 ± 0.037 and 0.362 ± 0.030, for SCC, SHS and PNS, respectively; while *h* ^2^ estimates using GRM_a were comparable for SCC (0.434 ± 0.037), and SHS (0.439 ± 0.038), and possibly lower for PNS (0.226 ± 0.032). Estimates of genetic correlation (*r*_g_) were similar for both models, except for the *r*_g_ between SHS and PNS which moved from −0.095 ± 0.119 using GRM_u to −0.298 ± 0.113 using GRM_a. For all traits, the correlation between GEBV from GRM_u and GRM_a was > 0.90, indicating similar ranking of animals regardless of the relationship matrix used. However, a random cross‐validation scheme showed that using GRM_a increased GEBV accuracies from 0.550 to 0.571 (or 3.7%) for SCC, from 0.496 to 0.509 (or 2.6%) for SHS and from 0.335 to 0.423 (or 26.2%) for PNS. Multi‐breed genomic predictions for tropical bull fertility are feasible alternative to individual single‐breed evaluation systems. Furthermore, the computational effort required to adjust the genomic relationship matrix for breed‐specific allele frequencies is justified by the significant improvement in prediction accuracy, ultimately enhancing the selection of bulls in tropical environments. Collectively these results enable very early in‐life selection for bull fertility traits, supporting genetic improvement strategies currently taking place within tropical beef production systems in northern Australia.

## 1 Introduction

Climate change adaptation in tropical beef systems requires genetic selection tools that enhance reproductive efficiency across diverse breeding environments. In northern Australia, the beef production system is largely extensive and relies heavily on tropical cattle grazing low quality, phosphorus‐deficient pastures with seasonal variations in nutritive value (Mwangi et al. 2022). Bull fertility is an important economic driver of profitability for beef cattle operations in tropical regions such as northern Australia. However, compared to female fertility, which has received much consideration and has been managed through the development of reproductive technologies (Bó and Baruselli 2014; Porto‐Neto et al. 2015; Baruselli et al. 2018) and early stage genomic selection (Zhang et al. 2014; Reverter et al. 2016; Hayes et al. 2019), many Australian tropical bull producers operate without formal genetic improvement programs, creating opportunities for the adoption of multi‐breed genomic prediction (GBLUP) models (Porto‐Neto et al. 2023). Working with a multi‐breed population of 3696 heifers in northern Australia, Hayes et al. (2019) determined that genomic evaluations for fertility in tropical beef cattle should be based on high‐density markers and should be multi‐breed. More recently, Menchon et al. (2024) concluded that while beef cattle producers in northern Australia preferred records of fertility, recording fertility data emerges as a barrier to the adoption of genetic tools.

Bull fertility characteristics are easier to assess than female traits and can be measured at an early age. Bulls typically reach puberty between 9 and 13 months, influenced by factors such as weight, breed, and nutrition (Rawlings et al. 2008). Key signs of pubertal development in bulls include rapid growth of the testicles and a higher count of normal sperm in an ejaculate (Rawlings et al. 2008). At present, scrotal circumference is the sole male trait assessed across all breeds for genetic evaluation, while the percentage of normal sperm is utilised in tropically adapted cattle to enhance herd reproduction (Graser et al. 2005; Corbet et al. 2013).

The variety of breeds used in tropical beef production systems of northern Australia restricts the ability to assemble the large reference populations needed for accurate genomic estimated breeding values (GEBV) for bull fertility. To overcome this challenge, strategies that integrate reference populations across breeds to produce accurate multi‐breed GEBV are currently being investigated (Hayes et al. 2023; Porto‐Neto et al. 2023; Warburton et al. 2023; Copley et al. 2024). However, most multi‐breed evaluations use a single genetic effect for all genotyped animals and a single genomic relationship matrix (GRM), resulting in the same single nucleotide polymorphism (SNP) effects for all breeds (Misztal et al. 2022). Since multi‐breed models should include SNP effects that account for breed differences, two suggested approaches include the use of meta founders (Legarra et al. 2015) and adjusting the GRM for breed‐specific allele frequencies (Lourenco et al. 2016; Wientjes et al. 2017). Being pseudo‐individuals acting as founders of the populations, meta founders have been recommended when combining pedigree and genomic information. Instead, adjusting the GRM for breed‐specific allele frequencies is particularly relevant in fully genotyped populations where no pedigree information is available.

In the present study, we hypothesise that adjusting the GRM for breed‐specific allele frequencies will improve prediction accuracy compared to the unadjusted GRM. Using an unpedigreed population of 2824 bulls from three tropically adapted breeds (Brahman (BRA), Santa Gertrudis (SGT) and Ultra Blak (UBL)), and focusing on three fertility traits (scrotal circumfersence (SCC), sheath score (SHS) and percent normal sperm (PNS)), we aim to compare the accuracy, bias and dispersion of GEBV resulting from five GBLUP models: three within breed and two multi‐breed. The latter contained a multi‐breed GRM computed without (GRM_u) or adjusting (GRM_a) for breed‐specific allele frequencies.

## 2 Materials and Methods

### 2.1 Population Details: Breeds, Phenotypes and Genotypes

Animals, phenotypes and genotypes used in the present study were a subset of those reported by Porto‐Neto et al. (2023). Specifically, we selected 2824 bulls from three tropically adapted breeds: Brahman (BRA; *N* = 1051), Santa Gertrudis (SGT; *N* = 929) and Ultra Black (UBL; *N* = 844); and focused on three fertility‐related phenotypes: scrotal circumference (SCC, cm) measured with a standard measuring tape, sheath score (SHS, score 1 to 5 for the SGT and UBL populations, and score 1 to 10 for the BRA population) and percent normal sperm (PNS, %) measured during the conduct of standard bull breeding soundness examination (BBSE; Fordyce et al. 2006). Table 1 shows the summary statistics for the age at measurement and three phenotypes by each breed.

**TABLE 1:** Summary statistics including number of records (*N*), mean, standard deviation (SD), minimum (Min.) and maximum (Max.) for the variables age, scrotal circumference (SCC), sheath score (SHS), and percent normal sperm (PNS) by breed.

Breed	Variable	*N*	Mean	SD	Min.	Max.
Brahman (BRA)	Age, d	1051	582.93	95.37	349	754
	SCC, cm	1051	27.93	2.80	20.50	40.50
	SHS, score	1051	5.89	1.12	3	9
	PNS, %	1051	51.57	30.24	0	98
Santa Gertrudis (SGT)	Age, d	929	626.14	34.56	537	757
	SCC, cm	918	34.46	3.10	25.5	52.5
	SHS, score	928	2.95	0.78	1.0	4.5
	PNS, %	895	73.36	21.44	0	99
Ultra Black (UBL)	Age, d	844	438.23	75.19	305	710
	SCC, cm	837	33.80	3.38	22.50	49.00
	SHS, score	842	1.78	0.80	1	4
	PNS, %	783	67.69	26.55	0	100

Most animals were genotyped using a commercial SNP chip with ~50 K markers. Genotypes were imputed up to high density (~700 K SNP) using a reference population genotyped on the higher density platform, mapped onto the ARS_UCD 1.2 cattle genome assembly (Rosen et al. 2020). Genotypes were first phased using Eagle (Loh et al. 2016) and then imputed using Minimac3 and Minimac4 for autosomes and the X chromosome, respectively (Das et al. 2016). SNP with imputation *r* ^2^ > 0.8 were kept for further analyses. In total, 680,758 SNP were kept including 24,058 mapped on the X chromosome. Based on this genotype information and numerical approaches outlined in Reverter et al. (2020) we estimated the genomic breed composition of the 2824 bulls in this study against three reference breeds: Angus, Brahman and Santa Gertrudis.

### 2.2 Genomic Prediction Models and Relationship Matrices

Before the genomic analyses, phenotypes were adjusted for non‐genetic effects using SAS 9.4 (SAS Institute Inc., Cary, NC, USA). As described in Porto‐Neto et al. (2023), the model for adjustment included the fixed effects of breed and contemporary group (CG) defined as year of birth and cohort (i.e., paddock), and the linear covariates of age at measurement and the first three principal components of the GRM (either GRM_u or GRM_a, depending on the analysis). The number of CG per breed was 45, 33 and 15 for BRA, SGT and UBL, respectively. Fitting the principal components aims at accounting for hidden population structures, likely with purebreds and crossbred populations, that could've been missed if only fitting CG.

The adjusted phenotypes were analysed using a fully random GBLUP model with additive polygenic and residual effects with assumed distributions N(**0**, G⨂V) and N(**0**, I⨂R), respectively, where **G** represents the GRM described next, **V** is the 3 × 3 (for as many traits) genetic co‐variance matrix, **I** is an identity matrix, **R** is the 3 × 3 (for as many traits) residual variance–covariance matrix and ⨂ represents the Kronecker product. In total, five tri‐variate GBLUP models were considered including three single‐breed and two multi‐breed:

1. SB_BRA: Single‐breed within Brahman (BRA) population.
2. SB_SGT: Single‐breed within Santa Gertrudis (SGT) population.
3. SB_UBL: Single‐breed within Ultra Black (UBL) population.
4. MB_GRM_u: Multi‐breed using an unadjusted GRM (GRM_u).
5. MB_GRM_a: Multi‐breed using a GRM adjusted for breed‐specific allele frequencies (GRM_a).

In all cases, variance components, heritability (*h* ^2^), genetic (*r*_g_) and residual (*r*_e_) correlations were estimated using the Qxpak5 software (Pérez‐Enciso and Misztal 2011). For the single‐breed models, as well as for the MB_GRM_u model, the GRM was obtained following Method 1 of VanRaden (2008):

$$\mathrm{GRM}=\frac{{\mathrm{ZZ}}^{T}}{2\sum p_{i}\left(1-p_{i}\right)},$$

where **Z** is the centred matrix relating SNP genotypes (recoded as 0, 1 or 2) in columns with animals in rows, and *p*_*i*_ is the frequency of the second allele of the *i*th SNP observed in the population under study, either single‐ or multi‐breed.

For the MB_GRM_a model, the GRM_a was obtained expanding derivations from Wientjes et al. (2017) to a 3‐breed scenario, with three blocks within a breed, and identical construction methods used for each block. However, allele frequencies were computed separately for each individual breed. The between‐breed blocks were obtained by multiplying the genotypes from two different breeds and dividing by the geometric mean of the sum of 2*p* (1–*p*) values from each breed. In detail, the GRM_a matrix was computed as follows:

$$\mathrm{GRM}\_a=\left[\begin{array}{ccc} G_{11} & G_{12} & G_{13}\\ G_{21} & G_{22} & G_{23}\\ G_{31} & G_{32} & G_{33} \end{array}\right]$$

$$=\left[\begin{array}{ccc} \frac{Z_{1}Z_{1}^{\prime }}{\sum 2p_{1j}\left(1-p_{1j}\right)} & \frac{Z_{1}Z_{2}^{\prime }}{\sqrt{\sum 2p_{1j}\left(1-p_{1j}\right)}\sqrt{\sum 2p_{2j}\left(1-p_{2j}\right)}} & \frac{Z_{1}Z_{3}^{\prime }}{\sqrt{\sum 2p_{1j}\left(1-p_{1j}\right)}\sqrt{\sum 2p_{3j}\left(1-p_{3j}\right)}}\\ \frac{Z_{2}Z_{1}^{\prime }}{\sqrt{\sum 2p_{1j}\left(1-p_{1j}\right)}\sqrt{\sum 2p_{2j}\left(1-p_{2j}\right)}} & \frac{Z_{2}Z_{2}^{\prime }}{\sum 2p_{2j}\left(1-p_{2j}\right)} & \frac{Z_{2}Z_{3}^{\prime }}{\sqrt{\sum 2p_{2j}\left(1-p_{2j}\right)}\sqrt{\sum 2p_{3j}\left(1-p_{3j}\right)}}\\ \frac{Z_{3}Z_{1}^{\prime }}{\sqrt{\sum 2p_{1j}\left(1-p_{1j}\right)}\sqrt{\sum 2p_{3j}\left(1-p_{3j}\right)}} & \frac{Z_{3}Z_{2}^{\prime }}{\sqrt{\sum 2p_{3j}\left(1-p_{3j}\right)}\sqrt{\sum 2p_{2j}\left(1-p_{2j}\right)}} & \frac{Z_{3}Z_{3}^{\prime }}{\sum 2p_{3j}\left(1-p_{3j}\right)} \end{array}\right],$$

where **Z**_1_, **Z**_2_ and **Z**_3_ represent the genotype matrices for breed 1, 2, and 3 (for BRA, SGT and UBL), respectively; *p*_1*j*_, *p*_2*j*_ and *p*_3*j*_ denote the frequencies of the A_2_ allele of the SNP j for breeds 1, 2, and 3, respectively; and the summation is across all SNP markers.

Before integrating GRM_u and GRM_a into the multi‐breed GBLUP models, a comparison was conducted to examine the distribution of their diagonal and off‐diagonal elements, in addition to being subjected to principal component analyses (PCA) to assess the proportion of variation explained by the leading principal components (PC) and to reveal breed‐specific clusters.

### 2.3 Cross‐Validation and the LR Method

For each of the five GBLUP tri‐variate models, the resulting GEBV were termed ${\hat{u}}_{w}$ to indicate that they are based on the *whole* dataset. Next, for the validation of genomic predictions, we developed a 5‐way 80/20 cross‐validation schema where at each round, a random 20% of the phenotypes were set as missing (validation set) and predicted from the remaining 80% (calibration set). In each cross‐validation schema, the resulting GEBV were termed ${\hat{u}}_{p}$ to indicate that they are based on *partial* data.

Finally, LR method (Legarra and Reverter 2018) approaches were used to estimate accuracy, bias, and dispersion of GEBV. The following three metrics were employed:

1. Accuracy (ACC): For individuals in the validation population, accuracy was computed as follows:
$$\mathrm{ACC}=\sqrt{\frac{\mathrm{cov}\left({\hat{u}}_{w},{\hat{u}}_{p}\right)}{\left(1+\bar{F}-2\bar{f}\right)\sigma_{g,\infty }^{2}}},$$
  where $\bar{F}$ is the average inbreeding coefficient obtained by subtracting one from the diagonal elements of the GRM, $2\bar{f}$ is the average relationship between individuals obtained from the off‐diagonal elements of the GRM, and $\sigma_{g,\infty }^{2}$ is the genetic variance at equilibrium in a population under selection. Assuming the individuals in the validation population are not under selection, $\sigma_{g,\infty }^{2}$ can be approximated by the additive genetic variance estimated from the partial dataset.
2. Bias: Difference between the average GEBV of individuals in the validation population using the partial data minus that using the whole data:
$${\text{Bias}}_{\mathrm{LR}}=\bar{{\hat{u}}_{p}}-\bar{{\hat{u}}_{w}}$$
  In the absence of bias, the expected value of Bias is 0; while positive and negative values indicate respectively overestimation and underestimation of GEBV for validation animals when their own observation was not included. To allow direct comparisons across traits, Bias was expressed in genetic standard deviation units by dividing the above expression by the square root of $\sigma_{g,\infty }^{2}$.
3. Dispersion: For individuals in the validation population, dispersion was measured from the slope of the regression of ${\hat{u}}_{w}$ on ${\hat{u}}_{p}$:
$$\text{Dispersion}=1-\frac{\mathrm{cov}\left({\hat{u}}_{w},{\hat{u}}_{p}\right)}{\mathrm{var}\left({\hat{u}}_{p}\right)}$$
  Although different to the traditional understanding of dispersion in the LR validation, our preference for using “1 − slope” arises from its more intuitive interpretation. In this new sense, the expected value of Dispersion is 0 in the absence of bias. Also, negative values indicate under‐dispersion (or deflation) of ${\hat{u}}_{p}$ into ${\hat{u}}_{w}$ as phenotypes become available, while positive values indicate over‐dispersion (or inflation) of ${\hat{u}}_{p}$ into ${\hat{u}}_{w}$.

## 3 Results and Discussion

### 3.1 Breeds, Phenotypes and Genotypes

As shown in Table 1, SGT bulls were on average measured at a later age (626.14 days) than either BRA (582.93 days) or UBL (438.23 days) bulls. That age difference could explain the larger values observed for SCC (34.46 cm) and PNS (73.36%) for SGT than for the other two breeds. However, the variation around the age at measurement in BRA and UBL was larger than in SGT, which may explain why PNS values > 95% were observed across the three populations (Table 1). Recently, Facy et al. (2024) examined BBSE traits on a population of 616 to 826 tropical composite bulls measured at around 12 months old and reported means (±SD) of 31.55 ± 4.05 cm, 1.59 ± 0.63 score, and 64.44% ± 26.32% for SCC, SHS, and PNS, respectively.

Table 2 confirms that the genomic breed composition of the three populations was, by and large, as nominated. BRA bulls were estimated to be, on average, 98.63% Brahman and ranging from 74.12% to 100%. The bull with the estimated Brahman component at 74.12% had its remaining 25.888% assigned to Santa Gertrudis. Meanwhile, SGT bulls were estimated to be 98.04% Santa Gertrudis on average, with one bull having as much as 18.99% Brahman. Finally, UBL bulls were estimated to be, on average, 73.32% Angus and 20.00% Brahman. These proportions agree with the expectations given that Ultra Black cattle are a crossbreed developed by crossing Brangus (a cross between Brahman and Angus) with Angus. Li et al. (2020) reported a population of Ultra Black animals which had on average 81.25% Angus composition.

**TABLE 2:** Genomic breed composition percentage, including mean, standard deviation (SD), minimum (Min.) and maximum (Max.) of the 2824 bulls examined in this study against three reference breeds: Angus, Brahman and Santa Gertrudis.

Nominal breed	Reference breed	Mean	SD	Min.	Max.
Brahman (*N* = 1051)	Angus	0.00	0.17	0.00	5.48
	Brahman	98.63	3.47	74.12	100.00
	Santa Gertrudis	1.25	3.27	0.00	25.88
Santa Gertrudis (*N* = 929)	Angus	0.02	0.36	0.00	7.05
	Brahman	0.78	2.48	0.00	18.99
	Santa Gertrudis	98.04	3.96	73.80	100.00
Ultra Black (*N* = 844)	Angus	73.32	12.33	38.79	100.00
	Brahman	20.00	9.22	0.00	54.73
	Santa Gertrudis	2.07	3.58	0.00	15.63

### 3.2 GRM_u vs. GRM_a

**FIGURE 1:**
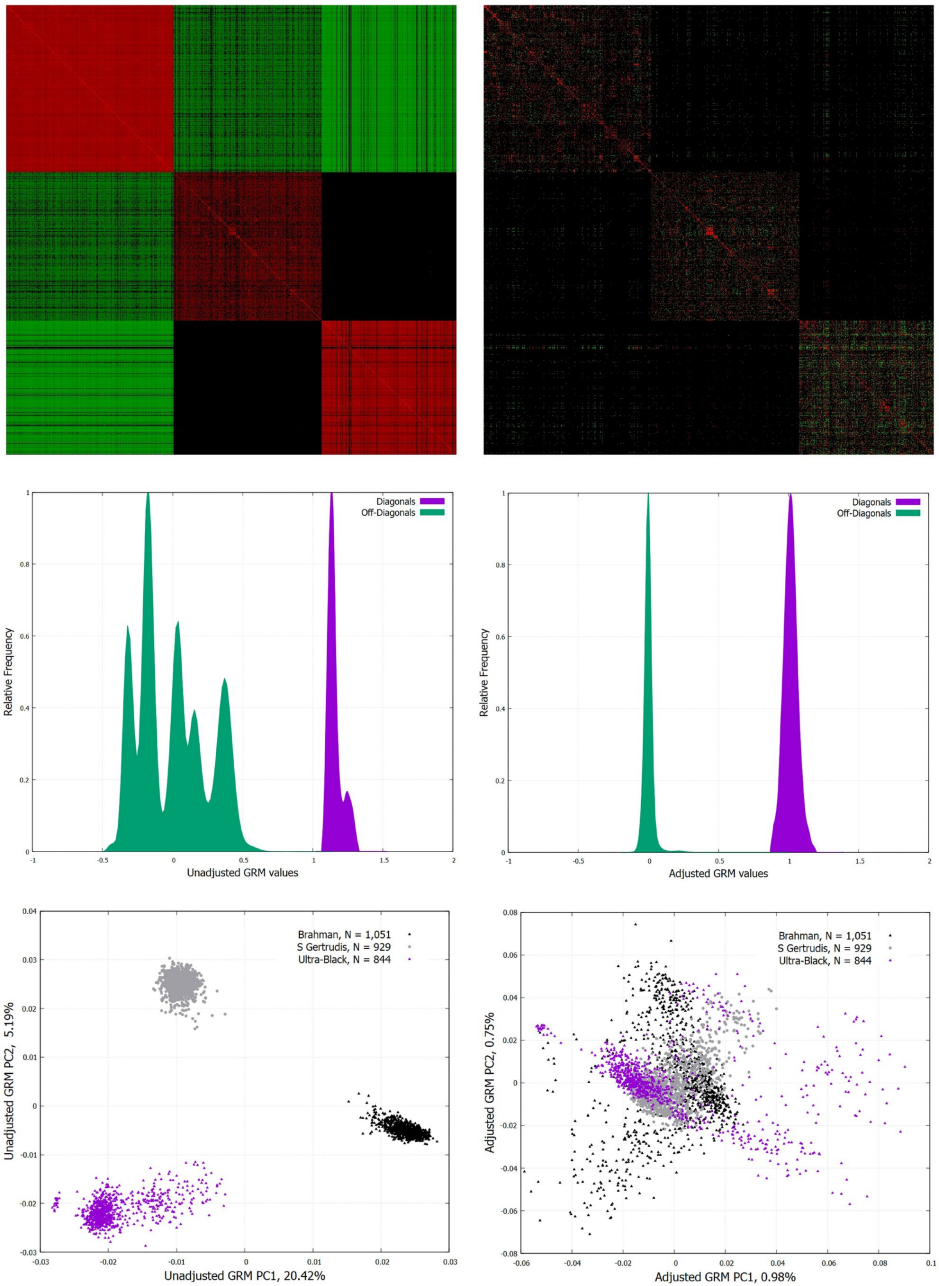
Multi‐breed genomic relationship matrices: Unadjusted (GRM_u; left panels) or adjusted (GRM_a; right panels) for breed specific allele frequencies. Top row: Heatmap of GRM_u (left) and GRM_a (right) with positive and negative values indicated by red and green, respectively. Middle row: Density distribution of the diagonals (green) and off‐diagonal values (purple) of GRM_u (left) and GRM_a (right). Bottom row: Scatter plot of the first (PC1; *x*‐axis) and second (PC2; *y*‐axis) principal components of GRM_u (left) and GRM_a (right) highlighting the three breed populations, Brahman (black dots), Santa Gertrudis (grey) and Ultra Black (purple). [Colour figure can be viewed at wileyonlinelibrary.com]

Figure 1 displays three sets of panels contrasting the unadjusted GRM_u against the breed‐adjusted GRM_a. Firstly, the heatmap of the GRM_u presents distinct blocks for each of the three breeds, BRA, SGT, and UBL, which show strong internal relationships (predominating red for positive values) and highly unrelated across blocks (predominating green for negative values), particularly notable for BRA in relation to the other two breeds. Instead, this breed‐specific blocks are nearly absent in the heatmap of the GRM_a, where only a few negative values (green) are visible, some within blocks, particularly for the last block associated with the UBL population. Secondly, the empirical distribution of diagonal (expected around one) and off‐diagonal (expected around zero) elements of the two GRMs show clear anomalies for GRM_u with several peaks including two within the negative range and likely for BRA relationships across the other two breed populations. On the other extreme, diagonal values of GRM_u were all positive with a major and a minor peak at around 1.15 and 1.25 respectively. Again, these anomalies were non‐existent in the GRM_a after adjusting for breed‐specific allele frequencies. Consistent with our results, Lourenco et al. (2016) concluded that using across‐breed allele frequencies when the correlations of allele frequencies between breeds differ from 1, could lead to genomic relationships between animals of different breeds that are on average negative; while using breed‐specific allele frequencies reduced the number of negative relationships between 2 pure breeds, pulling the average closer to 0.

Lastly, a PCA of GRM_u showed clear distinct clusters that correspond to each breed and with the first two principal components (PC1 and PC2), accounting > 25% of the variation in genomic relationships. Instead, the PCA of GRM_a did not uncover breed‐specific clusters with PC1 and PC2 explaining < 2% of the variation. We conclude that the breed‐specific allele frequencies adjustments performed in GRM_a have led to a homogeneous population from the perspective of genomic relationships. Principal components were used when adjusting phenotypes with the aim to account for population structure. However, fitting the principal components derived from the same GRM used in the GBLUP model can result in over‐correction, thereby diminishing predictive ability (Janss et al. 2012). We expect that the risk of over‐correction will be reduced when using GRM_a because its leading principal components account for much less variation compared to those derived from GRM_u.

While some studies have proposed analytical approaches for, and the effect of adjusting the GRM for breed‐specific allele frequencies on genetic parameter estimates and genomic predictions (Lourenco et al. 2016; Wientjes et al. 2017; Colombi et al. 2024; Ma et al. 2024; Londoño‐Gil et al. 2025), we could not find any study comparing the results of a PCA of the multi‐breed GRM before and after adjusting for breed‐specific allele frequencies. However, the original study of Porto‐Neto et al. (2023) where the data from the current study was sourced, included a PCA of the GRM of the entire population of 6063 bulls from six breeds. In that work, the first two principal components explained 15.8% of the variation. Similarly, the PCA of 4064 bulls from five Italian beef cattle breeds showed that 15.6% of the genetic variation was explained by the first two principal components (Colombi et al. 2024). The same two principal components explained 6% of the genomic variation in a population of 8316 Wagyu and Wagyu‐crossed cattle (Reverter et al. 2024). More recently, and on the other extreme of genomic variation, Reverter et al. (2025) using a selected population of 11,224 Australian Angus cattle showed that PC1 and PC2 accounted for just 3.25% of the variation in genomic relationships.

### 3.3 Genetic Parameter Estimates

**FIGURE 2:**
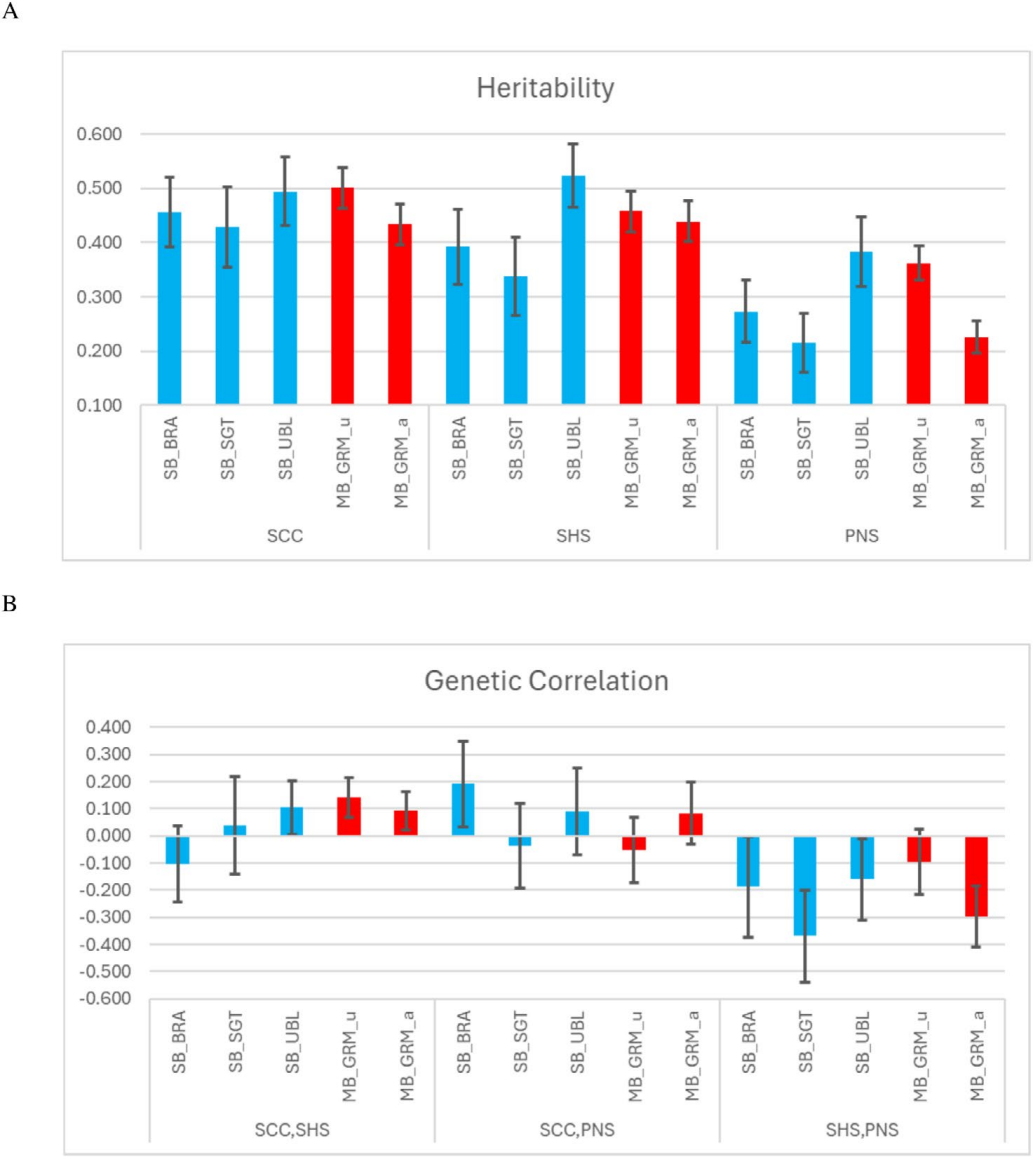
Estimates of heritability (A, top panel) and genetic correlation (B, bottom panel) for scrotal circumference (SCC), sheath score (SHS) and percent normal sperm (PNS) from the five genomic prediction models: three single‐breed (blue bars; Brahman (SB_BRA), Santa Gertrudis (SB_SGT) and Ultra Black (SB_UBL)) and two multi‐breed (red bars; MB_GRM_u and MB_GRM_a). Error bars represent standard errors. [Colour figure can be viewed at wileyonlinelibrary.com]

Figure 2A shows the estimated *h* ^2^ for the three traits across the five GBLUP models. For SCC, *h* ^2^ estimates were within one SE of each other and ranged from 0.428 ± 0.071 with SB_SGT to 0.501 ± 0.037 with MB_GRM_u. These estimates are very similar to the average estimate of 0.42 reported in the review of Berry et al. (2014), the 0.45 estimated from tropically adapted cattle by Facy et al. (2024), and the 0.47 reported in the original study of Porto‐Neto et al. (2023) where the data from the current study was sourced. Indeed, the literature is firm on the fact that SCC in bulls is at least moderately heritable and consequently would respond favourably to selection. For SHS, at 0.523 ± 0.058 the *h* ^2^ estimated with GBLUP model SB_UBL was comparatively higher than the one estimated with other single‐breed models, but within one SE of the ones estimated with the multi‐breed models. High *h* ^2^ estimates for SHS were also reported in the recent literature including the 0.49 of Facy et al. (2024) and the 0.57 of Porto‐Neto et al. (2023). The high *h* ^2^ estimate found with SB_UBL could be due to its categorical scale, as it is a subjective trait. Alternatively, it could also be due to the difference in SHS between breeds, because a tighter sheath is very breed specific (Table 1).

Finally, for PNS the *h* ^2^ estimates ranged from 0.215 ± 0.054 to 0.384 ± 0.065 for SB_SGT and SB_UBL, respectively. With the multi‐breed models, we observed a drop from 0.362 ± 0.032 with MB_GRM_u down to 0.226 ± 0.030 for MB_GRM_a. These estimates are lower than the 0.49 reported by Facy et al. (2024), but closer to the 0.24 reported by Porto‐Neto et al. (2023) or to the average of 0.222 ± 0.018 for sperm number reported in the review of Berry et al. (2014). In comparison, Fortes et al. (2020) reported a *h* ^2^ estimate for PNS of 0.35 and 0.29 for Brahman and tropical composite bulls.

Figure 2B shows the estimated *r*_g_ for the three pairs of traits across the five GBLUP models. The estimated *r*_g_ between SCC and SHS with the single‐breed models contained the value 0 in the ±1SE interval, while it was low positive for the multi‐breed models. For this pair of traits, comparable results were reported by Facy et al. (2024) and Porto‐Neto et al. (2023) with a *r*_g_ estimate of 0.20 and 0.24, respectively. A positive correlation between SCC and SHS is unfavourable as it indicates that similar genes affect the growth of both the scrotum and the sheath, which is important to consider in a breeding program. For the estimated *r*_g_ between SCC and PNS, the ±1SE interval contained zero with all GBLUP models except with SB_BRA where it was estimated at 0.192 ± 0.158. Our near zero estimates agree with the 0.05 estimated by Porto‐Neto et al. (2023) and the 0.02 estimated by Facy et al. (2024). However, positive (favourable from a breeding perspective) *r*_g_ between SCC and PNS have been reported including the 0.33 from Kealey et al. (2006) with Hereford bulls, and the 0.50 ± 0.13 from Corbet et al. (2013) with Brahman bulls. Consistent and negative (favourable) *r*_g_ were estimated between SHS and PNS. Among the single‐breed models, the strongest negative *r*_g_ was estimated with SB_SGT at −0.370 ± 0.168, while among the multi‐breed models, the most negative was obtained with MB_GRM_a at −0.298 ± 0.113. These values agree with the −0.26 estimated by Porto‐Neto et al. (2023) and the −0.33 estimated by Facy et al. (2024). Considering the adverse relationship between SCC and SHS, along with the almost negligible correlation between SCC and PNS, it could be argued that SHS serves as an ideal selection criterion for breeding programs focused on multi‐breed tropical bulls.

### 3.4 Genomic Predictions (GEBVs) and Cross‐Validation Results

Table 3 presents the Pearson correlation between GEBVs across the various GBLUP models and for the three traits analysed in this study. Indicating little re‐ranking of bulls, the multi‐breed models generated GEBVs that were very highly correlated with those from the single‐breed counterparts and ranged from 0.839 between SB_UBL and MB_GRM_u to 0.972 between SB_BRA and MB_GRM_u. The high correlations observed here could be attributed to a balance of numbers with no breed predominating. At 1051 bulls, our BRA population was only 13% larger than the SGT population (*N* = 929), which itself was only 10% larger than the UBL population (*N* = 844).

**TABLE 3:** Correlation between GEBV across the various GBLUP models^a^ and for the three traits analysed in this study: Scrotal circumference (SCC), sheath score (SHS), and percent normal sperm (PNS).

	SCC		SHS		PNS	
	MB_GRM_u	MB_GRM_a	MB_GRM_u	MB_GRM_a	MB_GRM_u	MB_GRM_a
SB_BRA	0.924	0.920	0.972	0.951	0.965	0.939
SB_SGT	0.930	0.944	0.935	0.944	0.916	0.887
SB_UBL	0.918	0.916	0.939	0.934	0.839	0.916
MB_GRM_u	1.000	0.993	1.000	0.973	1.000	0.912

^a^ SB_BRA: Single‐breed within Brahman (BRA) population; SB_SGT: Single‐breed within Santa Gertrudis (SGT) population; SB_UBL: Single‐breed within Ultra Black (UBL) population; MB_GRM_u: Multi‐breed using an unadjusted GRM (GRM_u); MB_GRM_a: Multi‐breed using a GRM adjusted for breed‐specific allele frequencies (GRM_a).

In situations where a single breed is over‐represented in the multi‐breed scenario, re‐ranking of animals from the less represented breeds is expected. Most recently, Londoño‐Gil et al. (2025) used a 4‐breed population of indicine Brazilian cattle including Nellore, Brahman, Guzerat, and Tabapua animals, where the number of records from Nellore cattle was larger than from the other three breeds combined. The authors reported more GEBV changes for the less representative breeds, with correlations ranging from 0.57 up to 0.81 for Brahman, 0.65 up to 0.86 for Guzerat, and 0.58 up to 0.69 for Tabapua, compared to the 0.99 up to 1.00 for Nellore.

It is worth noting that in all cases, single‐ and multi‐breed cross‐validation schemas, the phenotype exclusion was done on an individual animal basis. At each of the 5‐fold cross‐validation rounds, a random 20% of animals were assigned to the validation set and their measurements for the three traits removed. Should the exclusion phenotypes occur on a per record/trait basis, predictions for the masked trait in a given animal would benefit from the correlation with the other traits of the same animal and close relatives. Conversely, if the validation scheme in the multi‐breed analyses was to be stratified by breed, the accuracy of genomic predictions would be adversely affected.

**FIGURE 3:**
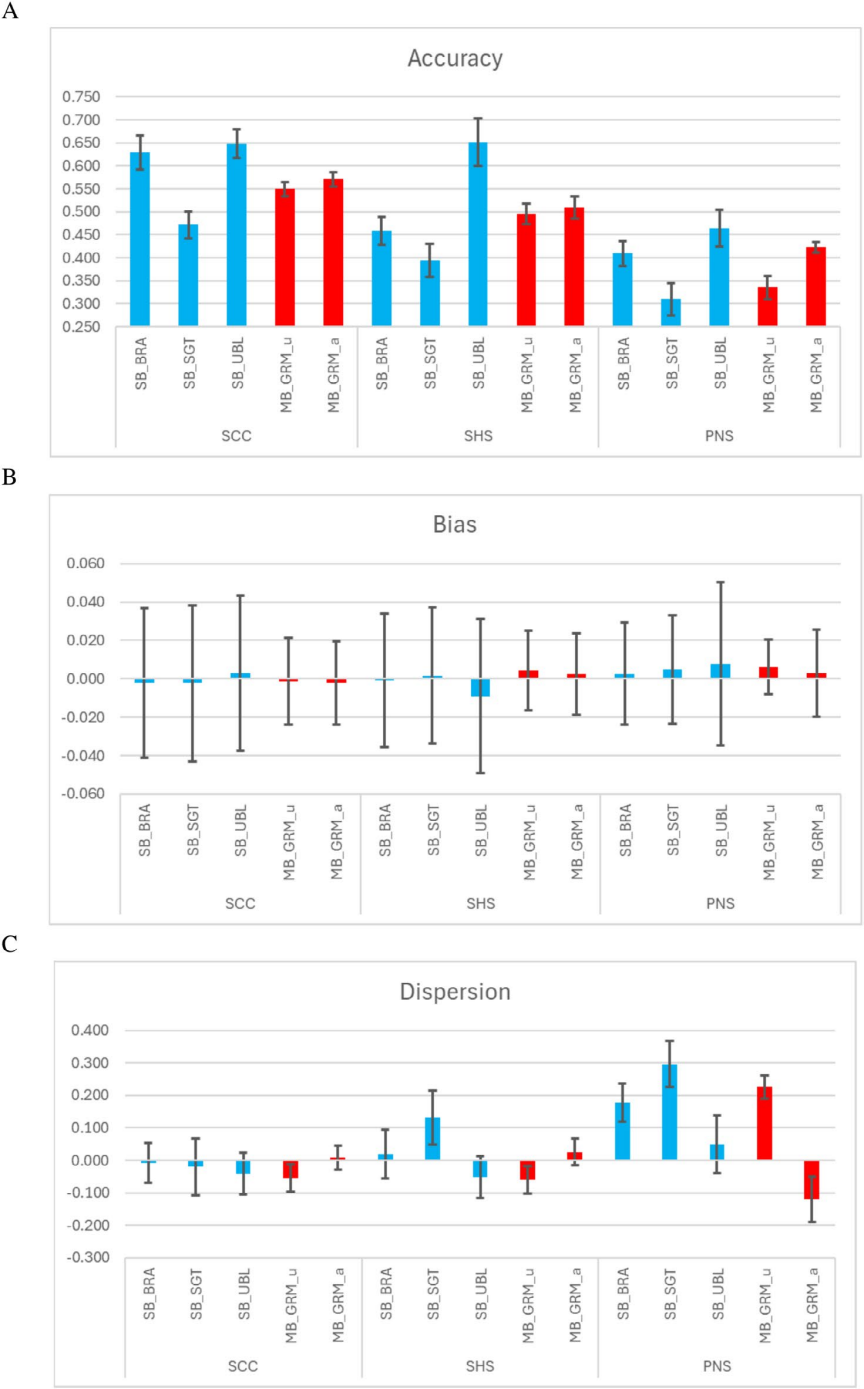
Genomic prediction accuracy (A, top panel), bias (B, middle panel) and dispersion (C. bottom panel) for scrotal circumference (SCC), sheath score (SHS) and percent normal sperm (PNS) from the five genomic prediction models: three single‐breed (blue bars; Brahman (SB_BRA), Santa Gertrudis (SB_SGT) and Ultra Black (SB_UBL)) and two multi‐breed (red bars; MB_GRM_u and MB_GRM_a). Error bars represent standard errors. [Colour figure can be viewed at wileyonlinelibrary.com]

Figure 3 shows the cross‐validation accuracy (Figure 3A, top panel), bias (Figure 3B, middle) and dispersion (Figure 3C, bottom) of GEBV computed for each GBLUP model and trait according to the LR method (Legarra and Reverter 2018). For SCC, the single‐breed GBLUP models showed GEBV accuracies of ~65% for both BRA and UBL populations, while for SGT, the accuracy of SB_SGT model was lower at 47.2% on average across the five cross‐validation splits. Instead, GEBV for SCC from the multi‐breed models had accuracies that were in the middle of the ones observed for single‐breed and valued at 0.550 ± 0.016 and 0.571 ± 0.015 for MB_GRM_u and MB_GRM_a, respectively. For SHS, the highest GEBV accuracy at 0.651 ± 0.051 was observed with SB_UBL, while the lowest at 0.394 ± 0.036 was observed with SB_SGT. Again, the multi‐breed models for SHS allowed for middle ground GEBV accuracies at ~50%. As acknowledged before, SHS results could be affected by measurement scale, as it is a subjective trait and breed specific. In fact, not only the scale, but also the coefficient of variation (CV) for SHS was markedly different across breeds, ranging from CV = 19.0% for BRA to CV = 45.1% for UBL, and possibly indicating the presence of heterogeneous residual variances which, if unaccounted, could impact genomic prediction accuracy. Based on simulation and real data, Ou et al. (2016) concluded that heteroskedastic error models showed enhanced prediction accuracy compared to homoskedastic error models, although the magnitude of the improvement was small and less than two percentage points net gain in prediction accuracy. Nonetheless, further research is needed to assess the potential accuracy gains by explicitly modelling residual variances as a function of various environmental or management factors such as, in our case, the scales for measuring SHS being different across breeds.

Finally, a similar pattern was observed for PNS, with single‐breed models with SGT and UBL populations having the lowest and highest GEBV accuracies, respectively. The accuracy values observed here with the MB_GRM_u model are in close agreement with those reported by Porto‐Neto et al. (2023) with an equivalent model. In that work, the authors estimated accuracies of 0.59, 0.49, and 0.35 for SCC, SHS, and PNS, respectively. However, for multi‐breed models and PNS, we observed a 26.2% increase in accuracy from MB_GRM_u (0.335 ± 0.025) to MB_GRM_a (0.423 ± 0.011), highlighting the importance of adjusting the GRM for breed‐specific allele frequencies in PNS analysis. This marked improvement for PNS likely reflects its lower heritability compared to SCC and SHS, making genomic predictions more sensitive to accurate relationship matrices.

Therefore, consistently among the single‐breed models and all three traits, the highest and lowest GEBV accuracy was observed for SB_UBL and SB_SGT, respectively; while the multi‐breed models generated GEBV accuracies that were, at all times, in the middle ground compared to those observed in the single‐breed models, and with GRM_a providing a moderate to large (certainly for PNS) increase in GEBV accuracy compared to GRM_u. Also consistent with expectations, across all models and traits, we observed a strong correlation (*r* = 0.830) between *h* ^2^ estimates (Figure 2A) and GEBV accuracies (Figure 3A). This is despite the MB_GRM_a models yielding lower *h* ^2^ estimates (certainly for PNS) and higher accuracies (certainly for PNS) than the MB_GRM_u counterparts.

The large increase in accuracy for PNS after adjusting the GRM despite the lower *h* ^2^ estimates deserves further discussion. Theory and experimental results have shown that, in addition to *h* ^2^, factors affecting genomic prediction accuracy include the size of the reference population (Daetwyler et al. 2008; Goddard 2009; Habier et al. 2010), relatedness between reference and validation population (de Roos et al. 2009; Wientjes et al. 2013), linkage disequilibrium (LD) between SNP markers and QTL (Habier et al. 2007; Wientjes et al. 2013), and the number of QTL (Daetwyler et al. 2010; Clark et al. 2011). In our present study, adjusting the GRM for breed‐specific allele frequencies has resulted in a more genomically homogeneous multi‐breed population (Figure 1), thereby creating the effect of a larger and more related reference population. Furthermore, the increase in accuracy is particularly significant for PNS, a trait reported to be controlled by a QTLs of large effect (Taylor et al. 2018; Modiba et al. 2022; Tan et al. 2024; Khan et al. 2024). It is therefore tempting to conclude that adjusting the GRM has resulted in better capturing the LD between the tagged SNP and those QTLs.

Regardless of trait and models, all GEBV were found to be unbiased (Figure 3B). However, reflecting the larger reference population, the error around the estimated biases was smaller for the multi‐breed models. For SCC, we found no signs of significant GEBV dispersion for any of the GBLUP models, but it was at its minimum for MB_GRM_a. For SHS, there were signs of overdispersion with the SB_SGT model, while again GEBV dispersion vanished with MB_GRM_a. Finally, GEBV for PNS was over‐dispersed for the single‐breed models SB_BRA and SB_SGT, as well as for the multi‐breed model MB_GRM_u. Few studies compare single‐ vs. multi‐breed models in terms of the dispersion of their resulting GEBV. Two exceptions include Londoño‐Gil et al. (2025) and Colombi et al. (2026) who found worse GEBV dispersion when the breeds were evaluated in the single‐breed approach, specially for the breeds with less data. Importantly, in our study and across the three traits considered, the multi‐breed model MB_GRM_a was the only model that showed dispersion of GEBV within the range of 15% considered as acceptable by the literature (Tsuruta et al. 2011; Bonifazi et al. 2022).

## 4 Conclusions

Climate change adaptation in tropical beef systems requires genetic selection tools that enhance reproductive efficiency across diverse, multi‐breed scenarios. Using a population of 2824 bulls from three tropically adapted breeds, we examined genomic predictions for three fertility traits and showed a consistent improvement in prediction accuracy using breed‐specific allele frequency adjustments. The present study highlights the potential of multi‐breed genomic models to generate predictions for tropical bull fertility that are on par with, or potentially superior to, those derived from single‐breed models, particularly for PNS. In our breed‐balanced dataset and for all three traits examined, there was little to no re‐ranking of bulls as we transitioned from single‐ to multi‐breed models. These findings confirm our hypothesis that adjusting the genomic relationship matrix for breed‐specific allele frequencies enhances prediction accuracy. The small to moderate increases in prediction accuracy warrant the computational effort required to adjust the genomic relationship matrix for breed‐specific allele frequencies in multi‐breed models. Our efforts support the adoption of sustainable bull breeding practices in tropical beef cattle operations.

## Funding

This research received internal strategic funding from CSIRO and external competitive funding from Meat & Livestock Australia.

## Conflicts of Interest

The authors declare no conflicts of interest.

## Data Availability

The data that supports this study may be shared upon reasonable request to the corresponding author.
